# Correction: Predictors of post-pericardiotomy syndrome after native valve-sparing aortic valve surgery

**DOI:** 10.1371/journal.pone.0317041

**Published:** 2024-12-31

**Authors:** Theresa Holst, Lisa Müller, Noureldin Abdelmoteleb, Sina Stock, Tatiana M. Sequeira Gross, Evaldas Girdauskas

There are errors in the Author Contributions. The correct contributions are:

**Conceptualization:** Theresa Holst, Evaldas Girdauskas

**Data curation:** Theresa Holst, Lisa Muller, Noureldin Abdelmoteleb, Sina Stock, Tatiana M. Sequeira Gross

**Formal analysis:** Theresa Hols**t**

**Investigation:** Theresa Holst

**Methodology**: Evaldas Girdauskas

**Project administration:** Evaldas Girdauskas

**Resources:** Evaldas Girdauskas

**Software:** Theresa Holst, Evaldas Girdauskas

**Supervision:** Evaldas Girdauskas

**Visualization:** Theresa Holst

**Writing—original draft:** Theresa Holst

**Writing—review & editing:** Lisa Muller, Noureldin Abdelmoteleb, Sina Stock, Tatiana M. Sequeira Gross, Evaldas Girdauskas.
